# High-intensity Focused Ultrasound Cycloplasty: Analysis of Pupil Dynamics

**DOI:** 10.5005/jp-journals-10028-1253

**Published:** 2018

**Authors:** David C Sousa, Nuno P Ferreira, Carlos Marques-Neves, Alix Somers, Evelien Vandewalle, Ingeborg Stalmans, Luís Abegão Pinto

**Affiliations:** 1 Department of Ophthalmology, Hospital de Santa Maria, Lisboa, PT, Portugal; 2 Vision Sciences Study Center, Faculdade de Medicina, Universidade de Lisboa, Lisboa, Portugal; 3 Department of Neurosciences, Research Group of Ophthalmology, KU Leuven, Leuven, Belgium; 4 Department of Ophthalmology, University Hospitals UZ Leuven, Leuven, Belgium

**Keywords:** Glaucoma, Intraocular pressure, Pupillometry, Ultrasound cystoplasty

## Abstract

**Aim:**

High-intensity focused ultrasound cystoplasty (UCP) aims to noninvasively and selectively target the ciliary body, thus lowering intraocular pressure (IOP). To be used on a large scale, the safety of the UCP procedure should be studied. Therefore, its effect on pupil behavior is important to better inform patients and to help physicians predict possible treatment side effects. This study aimed to evaluate to what extent UCP procedure (EyeOP-1®) affects pupil dynamics.

**Materials and methods:**

Consecutive glaucoma patients with uncontrolled IOP despite optimal medication scheduled for UCP treatment were recruited and followed for 6 months. Pupillometry (PlusoptiX^®^ S0_4_) was performed at baseline, and 1, 3 and 6 months after UCP procedure at scotopic and mesopic conditions. The difference between pupil diameter (PD) in both lighting conditions was calculated at the three follow-up visits. Demographic, clinical characteristics and specific ocular parameters (anterior chamber depth and volume, white-to-white measurement, axial length, phakic status) were registered. Statistical analysis was performed using STATA 14.1.

**Results:**

Sixteen eyes of 16 patients with a mean age of 69 ± 11 years were included. Mean preoperative IOP and number of medications were 23.6 ± 3.0 mm Hg and 2.4 ± 1.3, respectively. Mean baseline scotopic and mesopic PD were 4.8 ± 0.8 mm and 4.4 ± 0.9 mm, respectively (difference = 0.38 ± 0.30 mm; range 0.1 to 1.2 mm). At month-1, the pupil diameter (PD) change between scotopic (4.6 ± 0.7 mm) and mesopic (4.5 ± 0.8 mm) conditions decreased to 0.03 ± 0.34 mm, *p* = 0.01. On the longer follow-up periods, however, the amplitude difference in PD compared to baseline was no longer significant (month-3: 0.28 ± 0.49 mm; month 6: 0.23 ± 0.41 mm; *p* >0.05). At the end of follow-up, mean scotopic and mesopic PD were 4.7 ± 1.0 mm and 4.4 ± 0.9 mm, respectively.

**Conclusion and clinical significance:**

In the early postoperative period after UCP treatment, most patients present with a less light-reactive pupil, which seems to normalize with time.

**How to cite this article:**

Sousa DC, Ferreira NP, Marques-Neves C, Somers A, Vandewalle E, Stalmans I, Pinto LA. High-intensity Focused Ultrasound Cycloplasty: Analysis of Pupil Dynamics. J Curr Glaucoma Pract 2018;12(3):102-106.

## INTRODUCTION

Glaucoma is a leading cause of irreversible blindness, affecting more than 60 million people worldwide, and 50 million more by 2040.^1,2^ The increasing prevalence of a chronic disease constitutes a significant burden for the healthcare systems, along with the associated cost of life-long control visits, tests, and management of an aging population.^3–5^

It is estimated that approximately 20% of glaucoma patients are under three or more hypotensive classes, which may be considered an indication for surgery.^6,7^ Although traditional incisional surgery remains the gold-standard, the vision-threatening complications associated with the procedure and the post-operative care are limitations in real practice.^8,9^ Minimally invasive glaucoma surgery (MIGS) arose to help to cope with some of these limitations, consisting in a micro-invasive approach, reduced tissue trauma, at least moderate efficacy with a high safety profile, and a rapid recovery.^10,11^

In this context, a new technology, ultrasound cystoplasty (UCP), has been developed and increasingly used in practice.^12,13^ The UCP device consists of a circular probe with six transducers which use high-intensity focused ultrasound (HIFU) allowing for a noninvasive, reproducible and selective targeting of the ciliary body, thus preserving the surrounding healthy tissues.^12^ Several studies in early to moderate glaucoma have been published to date with encouraging results, reporting a mean IOP decrease of around 35% along with an 80% response rate.^13–19^

As most UCP studies have focused on standard outcomes in glaucoma surgical trials (i.e., IOP success and failure rates, need for re-intervention, change in visual acuity), its large-scale use is dependent on a better safety profile characterization. Since this device targets the ciliary body, its potential effects on iris function should be evaluated to rule out pupillary behavior changes which might cause glare or change in visual acuity.

This study aims to evaluate to what extent the UCP procedure affects pupil dynamics.

## MATERIALS AND METHODS

A prospective multicenter study was conducted in two tertiary ophthalmology clinics (Lisbon, PT, and Leuven, BE), from May 2016 to December 2017. The study protocol adheres to the tenets of the Declaration of Helsinki^20^ and was approved by both the Lisbon Academic Medical Center and Leuven University ethics’ committees. Written informed consent was obtained from each enrolled patient.

Consecutive open-angle adult glaucoma patients with uncontrolled IOP (> 20 mm Hg) despite optimal medication were scheduled to undergo UCP treatment. Participants underwent a full ophthalmic assessment including dynamic gonioscopy, IOP measurement using a Goldmann applanation tonometer, slit-lamp biomicroscopy, dilated fundus examination, and pupillometry.

Pupillometry (PlusoptiX^®^ S04, Plusoptix Inc., Atlanta, GA, USA) was performed at baseline (preoperatively), and 1, 3 and 6 months after UCP procedure. The pupillometry device uses an infrared eye-tracking that recorded horizontal and vertical pupil diameter (PD) and a high-resolution camera allowing to measure both pupils precisely. The participants were required to fixate on the center of the device, and during the examination, the pupil contours were monitored to assure a good-quality and reliable measurement. The pupillometry measurements were taken under two illumination levels–scotopic (0.1 cd/m^2^), and mesopic (1 cd/m^2^). In the darkness, after five minutes of darkness adaptation, three consecutive measurements were taken from each patient in scotopic and, then, in mesopic conditions. Horizontal and vertical PD were retrieved post-hoc from the device, and the average of the two was obtained. The PD difference between scotopic and mesopic conditions was used for evaluating pupil dynamics.

Demographic characteristics, ocular hypotensive drugs use and ocular parameters (anterior chamber depth and volume, white-to-white measurement, axial length, phakic status) were recorded to explore whether any of these were predictive for a specific pupillary change.

Exclusion criteria were history of previous filtering surgery, ocular or retrobulbar tumor, ocular infections or other diseases which may affect IOP assessment or cause ocular/systemic autonomic dysfunction (choroidal hemorrhage or detachment, lens subluxation, thyroid ophthalmopathy, proliferative diabetic retinopathy, clinically significant macular edema). Also, subjects that had a history of ocular trauma, uveitis, high myopia (> 6.0 D) or hyperopia (> 3.0 D) or known pupil abnormalities were excluded. To be included, patients also had to be cooperative enough to undergo pupillometry examinations.

Statistical analysis was performed using STATA v14.1, and *p* values < 0.05 were considered to indicate statistical significance. Data normality was assessed through Skewness-Kurtosis tests and histograms.

## RESULTS

Sixteen eyes of 16 patients (10 females) with a mean age of 69 ± 11 years were included. Mean preoperative IOP and number of medications were 23.6 ± 3.0 mm Hg and 2.4 ± 1.3, respectively. Mean baseline scotopic and mesopic PD were 4.8 ± 0.8 mm and 4.4 ± 0.9 mm, respectively (difference = 0.38 ± 0.30 mm; range 0.1 to 1.2 mm). Table 1 depicts the demographic and baseline data.

Mean preoperative IOP decreased to 13.1 ± 4.7 mm Hg (*p* < 0.001) at month 1, 14.4 ± 4.9 mm Hg (*p* < 0.001) at month 3, and 15.4 ± 5.9 mm Hg (*p* <0.001) at month 6. Considering an IOP decrease of at least 20% of baseline value (range: 20.7–29.7 mm Hg) and an IOP < 21 mm Hg, an 87.5% response rate was remarked, corresponding to a mean IOP reduction of 35%. The number of IOP-lowering medications remained similar to baseline for the first month and slightly reduced after 6 months to 2.1 ± 1.1 (*p* = 0.16) (Table 2).

**Graph 1:**
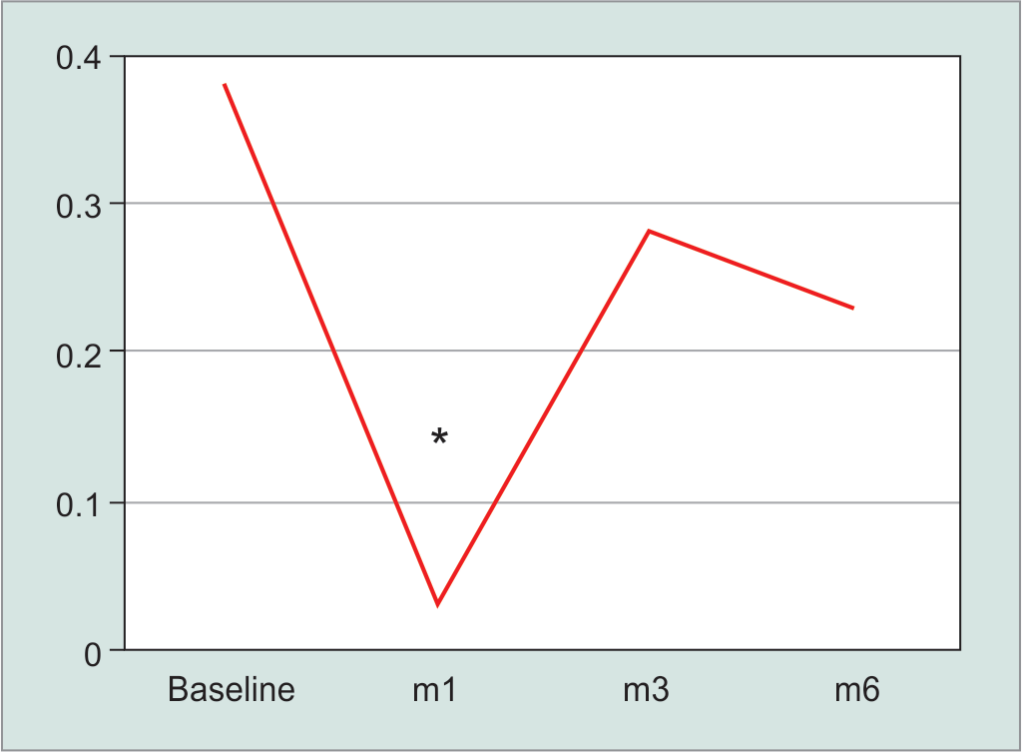
Graph depicting the change in pupil diameter during follow-up. Only at the very early post-operative period a statistically significant decrease in pupil diameter difference between scotopic and mesopic conditions was noted. *p = 0.01

At month 1, the PD difference between scotopic and mesopic conditions decreased to 0.03 ± 0.34 mm, *p* = 0.01 (Graph 1 and Table 2). This resulted from an increase in PD in mesopic conditions (Table 3). A similar change in vertical and horizontal PD was noted, suggesting a relatively similar treatment effect in superior and inferior quadrants. The vertical mean PD change decreased from 0.31 ± 0.41 mm at baseline to 0.06 ± 0.38 (*p* <0.05) at month 1. The horizontal mean PD change decreased from 0.29 ± 0.41 mm at baseline to 0.02 ± 0.22 (*p* <0.01) at month 1. Also, after one month, the vertical/horizontal ratio of the PD in scotopic and mesopic conditions was not significantly different comparing to baseline (both *p* = 0.07) (Table 3).

**Table 1:** Demographic and baseline data

*Male/ female,* n	*Age, years (mean ± SD range)*	*BCVA, logMar (mean ± SD)*	*IOP, mm Hg (mean ± SD [range])*	*Topical IOP drugs,* n *(mean ± SD)*	*Axial length, mm (mean ± SD)*	*Spherical equivalent, diopters (mean ± SD)*
6/10	69 ± 11 [42-82]	0.16 ± 0.21	23.6 ± 3.0 [20.7-29.7]	2.4 ± 1.3	23.5 ± 1.1	−0.1 ± 1.6
BCVA, best-corrected visual acuity, IOP, intraocular pressure, SD, standard deviation						

**Table 2:** Best-corrected visual acuity and pupil diameter changes during follow-up

	*BCVA, mm Hg*	*p*	*PD difference, mm Hg*	*p*	*IOP, mm Hg*	*p*	*Drops,* n	*p*
Baseline	0.16 ± 0.21	–	0.38 ± 0.30	–	23.6 ± 3.0	–	2.4 ± 1.3	–
Month 1	0.23 ± 0.19	0.07	0.03 ± 0.34	0.01	13.1 ± 4.7	< 0.001	2.4 ± 1.3	–
Month 3	0.19 ± 0.18	0.37	0.28 ± 0.49	0.31	14.4 ± 4.9	< 0.001	2.4 ± 1.3	–
Month 6	0.17 ± 0.21	0.63	0.23 ± 0.41	0.16	15.4 ± 5.9	< 0.001	2.1 ± 1.1	0.16
BCVA, best-corrected visual acuity, IOP, intraocular pressure, PD, mean pupil diameter, SD, standard deviation. PD difference was calculated as the mean scotopic PD minus mean mesopic PD. *p* values are considered versus baseline								

In longer follow-up periods, the amplitude in PD between scotopic and mesopic conditions was not different than baseline values (month-3: 0.28 ± 0.49 mm; month 6: 0.23 ± 0.41 mm; both *p* >0.05) (Fig. 1 and Table 2).

The univariate and multivariate analysis revealed that none of the studied ocular parameters (anterior chamber depth and volume, white-to-white distance, axial length, and phakic status) were associated with an increased chance of an altered pupil response. Moreover, the efficacy of treatment (i.e., IOP reduction) was also not associated with the magnitude of pupillary changes.

## DISCUSSION

This study aimed to characterize the possible changes in the pupillary behavior after UCP. Our results disclosed a statistically significant decrease in PD amplitude at 1-month follow-up when compared to preoperative values. As this change was mostly attributable to an increased PD in mesopic conditions, these findings suggest a relative limitation of iris reactivity to light in the early postoperative period. However, these were noted to be transient, being statistically significant only at month-1, with a return to near-baseline values during follow-up. UCP technology was developed and studied extensively *in vitro*. By using HIFU, the eye-adapted miniaturized device targets very selectively the ciliary processes epithelium in the 6 treated sectors (three superiorly and three inferiorly), without damaging the adjacent ocular structures.^17^ This treatment selectivity contributes to the rapid recovery and minimal inflammation observed in the post-operative period.

Although transient, it is important to understand the etiology of the pupil abnormalities in the early post-operative period. A possible mechanism would be a mechanism similar to Urrets-Zavalia syndrome, described originally in 1963 after penetrating keratoplasty.^21^ It consists in a fixed and dilated pupil after surgery, and since then it has been associated to other procedures such as lamellar keratoplasty, intracameral gas injection, trabeculectomy, goniotomy, cataract surgery and transscleral laser photocoagulation.^22–27^

**Table 3:** Scotopic and mesopic vertical and horizontal pupil diameter changes during follow-up

	*Scotopic*		*Mesopic*		*PD Amplitude*		*Vertical/Horizontal PD ratio*	
	*V*	*H*	*V*	*H*	*V*	*H*	*Scot*	*Mesop*
Baseline	4.4 ± 1.0	4.9 ± 0.7	4.1 ± 1.1	4.4 ± 1.0	0.31 ± 0.41	0.29 ± 0.41	0.90 ± 0.06	0.90 ± 0.06
Month 1	4.5 ± 0.7	4.8 ± 0.7	4.4 ± 0.8	4.8 ± 0.7	0.06 ± 0.38*	0.02 ± 0.22**	0.94 ± 0.04§	0.93 ± 0.07§
Month 3	4.6 ± 0.7	5.2 ± 0.8	4.5 ± 0.9	4.8 ± 0.8	0.19 ± 0.44	0.44 ± 0.56	0.89 ± 0.09	0.94 ± 0.05
Month 6	4.6 ± 1.0	5.2 ± 1.0	4.3 ± 1.1	4.7 ± 1.1	0.23 ± 0.37	0.34 ± 0.50	0.90 ± 0.08	0.91 ± 0.06
PD, mean pupil diameter; H, horizontal diameter; V, vertical diameter; Mesop, V/H PD ratio in mesopic conditions; Scot, V/H PD ratio in scotopic conditions. Mean values and corresponding standard deviations are depicted. PD amplitude was calculated as “scotopic vertical PD minus mesopic vertical PD” and “scotopic horizontal PD minus horizontal PD”, respectively. Vertical/Horizontal PD ratio was calculated as “scotopic vertical PD divided by scotopic horizontal PD” and “mesopic vertical PD divided by mesopic horizontal PD”, respectively *p* values are considered versus baseline: * *p* < 0.05, ** *p* = 0.01, § *p* = 0.07.								

However, the original description of the syndrome attributes the features to sphincter muscle ischemia and iris atrophy as a consequence from postoperative IOP spikes. While there may be a moderate and short-lasting increase in IOP during the UCP procedure related to the relatively low-level suction needed to help to stabilize the device,^18^ the outcome IOP is at least 30% lower than baseline values and iris atrophy is not noted on transillumination at slit-lamp during follow-up. Therefore, it would seem that intra-operative increases in IOP may not fully explain this phenomenon. We hypothesize 2 further mechanisms for the iris behavior changes encountered after UCP treatment. Firstly, normal anatomical variations of the ciliary processes distance to limbus exist. To adjust for these variations of eye anatomy, three UCP probe sizes have been developed. Biometric parameters such as axial length and white-to-white corneal diameter are used to select the most appropriate probe size to target as selectively as possible the ciliary processes. However, these normal individuals and sometimes unpredictable anatomical variations may explain the pupillary changes if the treated area reaches even slightly the peripheral iris. The preoperative use of pilocarpine may theoretically reduce its probability of occurrence and further comparative studies with or without pilocarpine use should be pursued to clarify this hypothesis. Secondly, the parasympathetic postganglionic short ciliary nerves run in a radial pattern and penetrate the sclera to reach the ciliary muscle and iris. Although the UCP device spares the 3- and 9-o'clock positions, a possible treatment effect in these small nerves may cause the changes observed in the pupil diameter. A pupillary dysfunction after surgery does not seem to be an indicator of correct or incorrect treatment, but instead an individual and somewhat unpredictable response to UCP. Our findings and its interpretation should be considered against the limitations of the present study. Firstly, since all the patients had glaucoma, inherent iris function abnormalities may have been present at baseline and introduce bias during follow-up evaluations and analysis. Secondly, as the objective changes in pupil behavior were the aim of this study, patient-related outcome measurements were not considered in detail. It would be interesting to relate objective changes with complaints such as photophobia, glare or decreased visual acuity. Also, although topical IOP-lowering drug classes were not statistically significantly associated with the PD changes, the fact that the patients were under different classes with some known to affect pupillary function may contribute as potential confounder as well. And lastly, as a pilot study, the sample size was relatively small which limits the statistical power on regression analysis and the possibility of further considerations on predictors of this iris behavior.

## CONCLUSION AND CLINICAL SIGNIFICANCE

The UCP can be associated with mostly transient alterations in pupil dynamics. The majority of patients presented with a reduction in pupil light-reactivity in the early postoprative period, which disappeared within 3 months postoperatively.
